# Social Determinants of Health and Delivery of Rehabilitation to Older Adults During ICU Hospitalization

**DOI:** 10.1001/jamanetworkopen.2024.10713

**Published:** 2024-05-10

**Authors:** Snigdha Jain, Terrence E. Murphy, Jason R. Falvey, Linda Leo-Summers, John R. O’Leary, Emma Zang, Thomas M. Gill, Harlan M. Krumholz, Lauren E. Ferrante

**Affiliations:** 1 Section of Pulmonary, Critical Care, and Sleep Medicine, Department of Internal Medicine, Yale School of Medicine, New Haven, Connecticut; 2 Department of Public Health Sciences, Pennsylvania State University, State College; 3 Department of Physical Therapy and Rehabilitation Science, University of Maryland School of Medicine, Baltimore; 4 Program on Aging, Yale School of Medicine, New Haven, Connecticut; 5 Department of Sociology, Yale University, New Haven, Connecticut; 6 Center for Outcomes Research and Evaluation, Yale New Haven Hospital, New Haven, Connecticut

## Abstract

**Importance:**

Older adults with socioeconomic disadvantage develop a greater burden of disability after critical illness than those without socioeconomic disadvantage. The delivery of in-hospital rehabilitation that can mitigate functional decline may be influenced by social determinants of health (SDOH). Whether rehabilitation delivery differs by SDOH during critical illness hospitalization is not known.

**Objective:**

To evaluate whether SDOH are associated with the delivery of skilled rehabilitation during critical illness hospitalization among older adults.

**Design, Setting, and Participants:**

This cohort study used data from the National Health and Aging Trends Study linked with Medicare claims (2011-2018). Participants included older adults hospitalized with a stay in the intensive care unit (ICU). Data were analyzed from August 2022 to September 2023.

**Exposures:**

Dual eligibility for Medicare and Medicaid, education, income, limited English proficiency (LEP), and rural residence.

**Main Outcome and Measures:**

The primary outcome was delivery of physical therapy (PT) and/or occupational therapy (OT) during ICU hospitalization, characterized as any in-hospital PT or OT and rate of in-hospital PT or OT, calculated as total number of units divided by length of stay.

**Results:**

In the sample of 1618 ICU hospitalizations (median [IQR] patient age, 81.0 [75.0-86.0] years; 842 [52.0%] female), 371 hospitalizations (22.9%) were among patients with dual Medicare and Medicaid eligibility, 523 hospitalizations (32.6%) were among patients with less than high school education, 320 hospitalizations (19.8%) were for patients with rural residence, and 56 hospitalizations (3.5%) were among patients with LEP. A total of 1076 hospitalized patients (68.5%) received any PT or OT, with a mean rate of 0.94 (95% CI, 0.86-1.02) units/d. After adjustment for age, sex, prehospitalization disability, mechanical ventilation, and organ dysfunction, factors associated with lower odds of receipt of PT or OT included dual Medicare and Medicaid eligibility (adjusted odds ratio, 0.70 [95% CI, 0.50-0.97]) and rural residence (adjusted odds ratio, 0.65 [95% CI, 0.48-0.87]). LEP was associated with a lower rate of PT or OT (adjusted rate ratio, 0.55 [95% CI, 0.32-0.94]).

**Conclusions and Relevance:**

These findings highlight the need to consider SDOH in efforts to promote rehabilitation delivery during ICU hospitalization and to investigate factors underlying inequities in this practice.

## Key Points

**Question:**

Are social determinants associated with differential delivery of skilled rehabilitation services to older adults during hospitalization with a critical illness?

**Findings:**

In this cohort study of 1618 hospitalizations in older adults, after accounting for prehospitalization disability and acute illness characteristics, dual Medicare and Medicaid eligibility and rural residence were associated with a lower likelihood of delivery of any rehabilitation, whereas limited English proficiency was associated with reduced amount of rehabilitation services delivered during a critical illness hospitalization.

**Meaning:**

These findings suggest social determinants of health should be taken into consideration in efforts to enhance equitable delivery of skilled rehabilitation to older adults who are critically ill.

## Teaser

This cohort study assesses the association between social determinants of health and receipt of skilled rehabilitation during intensive care unit (ICU) hospitalizations for older US adults.

## Introduction

Surviving a critical illness, an increasingly common occurrence among older adults,^1,2,3^ is frequently accompanied by new or worsening disability.^4,5^ Skilled rehabilitation with physical therapy (PT) and occupational therapy (OT) during hospitalization facilitates mobilization of patients recovering from critical illness and is known to prevent functional decline ^6,7,8^ and help to identify post-acute care needs.^9^ Therefore, inequitable delivery of rehabilitation services to older adults with social or economic disadvantage may contribute to downstream disparities in disability.^10^ Whether social determinants of health (SDOH) are associated with differences in delivery of skilled rehabilitation during a critical illness hospitalization is unknown.

Prior studies have described wide variation in clinician-reported delivery of rehabilitation services to patients who are critically ill.^11,12,13^ Cross-sectional studies of hospitals participating in trial networks or quality improvement collaboratives have yielded similar results.^14,15,16^ However, these studies did not investigate associations between SDOH and delivery of in-hospital rehabilitation. Many patient- and hospital-level factors known to be associated with prescription of skilled rehabilitation to patients who are critically ill can be affected by SDOH.^11,17,18,19^ For example, limited English proficiency (LEP) could be associated with lower rehabilitation delivery due to perceived barriers to engagement with therapy services or differences in management of sedation and delirium by clinicians.^20^ Furthermore, variation in resources and practices at hospitals caring for a higher proportion of patients with socioeconomic disadvantage could lead to reduced delivery of skilled rehabilitation services, as has been observed for other care processes.^21,22^ A common challenge in evaluation of health care disparities is the absence of granular information on SDOH, such as LEP, income, and education, and preexisting health status, that could influence in-hospital treatment needs, such as prehospitalization disability in the case of rehabilitation services. We leveraged a nationally representative longitudinal study of aging with detailed information on SDOH and prehospitalization geriatric risk factors, linked with administrative claims, to investigate whether SDOH are associated with differences in the delivery of skilled rehabilitation to older adults during hospitalization in an intensive care units (ICU).

## Methods

The protocol for the National Health and Aging Trends Study (NHATS) was approved by the Johns Hopkins University institutional review board, and our cohort study using these data was approved by the Yale University institutional review board. All participants provided informed consent. We followed the Strengthening the Reporting of Observational Studies in Epidemiology (STROBE) reporting guideline.

### Study Population

Data were drawn from the National Health and Aging Trends Study (NHATS), a longitudinal, nationally representative survey of community-dwelling Medicare beneficiaries ages 65 years and older living in the contiguous United States.^23^ The initial sample was drawn from the Medicare enrollment database on September 30, 2010.^24^ The survey collected information on sociodemographics, including race and ethnicity, insurance, education, income, English proficiency, rural residence, and clinical characteristics, through annual in-person interviews starting in 2011. If a participant was unavailable for interview, a proxy knowledgeable about their health was interviewed. Race and ethnicity were categorized as Hispanic, non-Hispanic Black, non-Hispanic White, and other (including participants reporting race as American Indian, Alaska Native, Asian, Native Hawaiian, Pacific Islander, or other race). Race and ethnicity were included in descriptive analyses because they are key SDOH.

### Ascertainment of ICU Admissions and Acquisition of ICU Hospitalization Data

ICU admissions were identified through linked inpatient claims files for Medicare fee-for-service and Medicare Advantage participants using critical care revenue codes indicating admission to general, specialty, or coronary care units but excluding psychiatric and intermediate care units.^25^ Information on mechanical ventilation and organ dysfunction was obtained using *International Classification of Diseases, Ninth Revision, Clinical Modification *(*ICD-9-CM*) and* International Statistical Classification of Diseases, Tenth Revision, Clinical Modification *(*ICD-10-CM*) diagnosis and procedure codes (eTable 1 in Supplement 1).^26,27^ ICU length of stay was determined based on days with a critical care revenue code.

### Outcome Ascertainment

Our primary outcomes were delivery of any PT or OT, as determined by revenue center codes 042.X and 043.X respectively, and amount of PT or OT, determined as number of units of evaluation or treatment delivered during ICU hospitalization. In general, PT and OT are billed in 15-minute increments; therefore, 1 billed unit represents 15 minutes of intervention by a therapist. These units were modeled as units per day to account for differences in hospital length of stay. Since services between 8 and 22 minutes are aggregated as a single unit, the observed rate of therapy is a rounded assessment of the actual delivered amount of therapy.

### Assessment of SDOH

Given its previously reported associations with increased risk for functional decline following critical illness and reduced delivery of in-hospital rehabilitation to older adults, our primary exposure was dual eligibility for Medicare and Medicaid.^10,28^ We additionally explored associations of other SDOH that have been linked to rehabilitation delivery in other populations or settings^29,30,31^ and were available in NHATS or Medicare claims data. SDOH assessed in this study included income, education, LEP, and rurality. Dual-eligibility for Medicare and Medicaid was assessed using the dual Medicare-Medicaid status indicator in the Medicare Master Beneficiary Summary File at any time during the year preceding the ICU hospitalization. Information on other SDOH was derived from the participant’s NHATS survey immediately preceding ICU hospitalization. Income and assets were assessed using a composite of income from Social Security; Department of Veterans Affairs; pension; retirement plans; funds, stocks, and bonds; and checking and savings accounts and operationalized as quartiles in our sample.^23^ The only missing data were on household income; missing data were imputed using values provided by NHATS.^32^ Education was characterized as less than high school vs more. LEP was operationalized as a response of not well or not at all, as opposed to well or very well, to questions about how well respondents understand or speak English.^23,33^ Residence was classified as rural (nonmetropolitan) vs urban (metropolitan), as assigned by NHATS based on the Office of Management and Budget classification of county of residence. We considered but did not evaluate the exposure of race and ethnicity because of small proportions of participants who identified as a race other than Black or White in our sample.

### Assessment of Covariates

We selected covariates that could be potential confounders in rehabilitation delivery based on prior research and clinical relevance. We included age categorized into intervals based on proportions in our sample (65-74, 75-79, 80-84, 85-89, and ≥90 years), sex, count of disabilities in the NHATS interview preceding ICU hospitalization (defined as need for help or inability to perform activities of daily living, including 4 self-care activities [eating, bathing, using the toilet, and dressing] and 3 mobility activities [getting outside, getting around inside one’s home, and getting out of bed]),^10,34^ use of mechanical ventilation (eTable 1 in Supplement 1), and severity of illness (determined as count of organ dysfunction).^27^

### Assembly of the Analytic Sample

Assembly of the analytic sample is presented in the eFigure in Supplement 1. We identified 2832 NHATS participants from 2011 to 2018 who had a hospitalization with an ICU admission for at least 1 day. Participants could contribute multiple observations; however, we restricted our sample to 2299 first ICU hospitalizations in the interval between consecutive annual NHATS interviews to allow updating model covariates. After excluding hospitalization from 681 participants who were not community-dwelling at the pre-ICU NHATS interview, our sample included 1618 ICU hospitalizations.

### Statistical Analysis

We describe demographic and clinical characteristics of our sample using means and SDs or medians and IQRs for continuous variables and counts and weighted percentages for categorical variables, as appropriate. For each person-year of NHATS data, we used specific analytic weights that adjust for differential probabilities of selection and nonresponse within each strata (region) and cluster (zip code within county); this allows generalization to the 2011 Medicare population.^35,36^ For income, NHATS provided 5 imputed data sets that were used only in the models testing this exposure.^32^ Among other exposures considered in the models, only education had any missing data (0.7%). Hence, our models were based on complete case data. We separately fit multivariable logistic regression models for the binary outcome of delivery of any in-hospital PT or OT on each of the 5 exposures with adjustment for covariates. For exposures with a significant association with this outcome, we calculated risk differences. For the rate of PT or OT delivered per day of hospital stay, we fit multivariable Poisson regression models on each of the exposures with adjustment for the same covariates. We calculated least square means of the outcomes significantly associated with this outcome. Because the provision of PT or OT could be prioritized to patients presumed to be discharging to a facility, we conducted sensitivity analyses excluding participants admitted from a nursing home or with a stay 100 days or more between pre-ICU NHATS interview and index ICU hospitalization. We used SAS software version 9.4 (SAS Institute) for descriptive analyses and SAS-callable SUDAAN software version 11 (RTI International) for all models. To account for the small number of participants who contributed multiple hospitalizations, we used generalized estimating equations with an exchangeable covariance structure based on its minimization of quasilikelihood under the independence model criterion. In all analyses, significance was defined as a 2-tailed *P* < .05. Data were analyzed from August 2022 to September 2023.

## Results

Our sample included 1618 ICU hospitalizations across 569 hospitals (Table). Patients had a median (IQR) age of 81.0 (75.0-86.0) years, and 842 (52.0%) were female. The sample included 371 patients (22.9%) with dual Medicare and Medicaid eligibility, 523 patients (32.6%) with less than high school education, 320 patients (19.8%) with rural residence, and 56 patients (3.5%) with LEP. Median (IQR) income was $22 000 ($12 000-$41 000).

**Table.** Characteristics of ICU Hospitalizations Overall, and by Delivery of PT or OT^a^

Characteristic	Hospitalizations, No. (%)		
	Overall cohort	Any PT or OT delivered	No PT or OT delivered
Sample, No.	1618	1076	542
Weighted, No	9 595 455	6 573 048	3 022 407
Age, y			
Median (IQR)	81.0 (75.0-86.0)	82.0 (76.0-87.0)	80.0 (74.0-85.0)
65-74	340 (21.0)	203 (18.9)	137 (25.3)
75-79	345 (21.3)	228 (21.2)	117 (21.6)
80-84	391 (24.2)	253 (23.5)	138 (25.5)
85-89	318 (19.6)	226 (21.0)	92 (17.0)
≥90	224 (13.8)	166 (15.4)	58 (10.7)
Sex			
Male	776 (48.0)	511 (47.5)	265 (48.9)
Female	842 (52.0)	565 (52.5)	277 (51.1)
Race and ethnicity^b^			
Hispanic	87 (5.4)	51 (4.8)	36 (6.7)
Non-Hispanic Black	401 (25.0)	260 (24.4)	141 (26.1)
Non-Hispanic White	1071 (66.8)	719 (67.6)	352 (65.2)
Other	45 (2.8)	34 (3.2)	11 (2.0)
Dual Medicare and Medicaid eligibility	371 (22.9)	228 (21.2)	143 (26.4)
Income, median (IQR), $	22 000 (12 000-41 000)	24 000 (12 268-45 000)	20 266 (12 000-40 000)
Chronic conditions, median (IQR), No.^c^	3.0 (2.0-4.0)	3.0 (2.0-4.0)	3.0 (2.0-4.0)
<High school education	523 (32.6)	337 (31.6)	186 (34.4)
Rural residence	320 (19.8)	185 (17.2)	135 (24.9)
Limited English proficiency	56 (3.5)	34 (3.2)	22 (4.1)
Frailty, median (IQR)^d^	2.0 (1.0-3.0)	2.0 (1.0-3.0)	2.0 (1.0-3.0)
Count of disabilities, median (IQR)^e^	0.0 (0.0-2.0)	0.0 (0.0-2.0)	0.0 (0.0-2.0)
Probable dementia	324 (20.0)	217 (20.2)	107 (19.7)
ICU length of stay, median (IQR), d^f^	2.0 (1.0-4.0)	2.0 (1.0-3.0)	2.0 (1.0-5.0)
Discharge diagnoses			
Cardiovascular disorders	531 (32.8)	313 (29.1)	218 (40.2)
Infectious disorders including pneumonia	275 (17)	188 (17.5)	87 (16.1)
Neurological disorders	230 (14.2)	181 (16.8)	49 (9.0)
Gastrointestinal disorders	162 (10.0)	102 (9.5)	60 (11.1)
Respiratory disorders	127 (7.8)	85 (7.9)	42 (7.8)
Mechanical ventilation^f^^,^^g^	256 (15.8)	168 (15.6)	88 (16.2)
Count of organ dysfunction, median (IQR)^f^	1.0 (0.0-1.0)	1.0 (0.0-1.0)	0.0 (0.0-1.0)

Abbreviations: ICU, intensive care unit; OT, occupational therapy; PT, physical therapy.

^a^ Values represent characteristics for the unweighted sample. For income, imputed data sets provided by NHATS were used. Among the remaining variables, missingness was 0.8% for race and ethnicity and 0.7% for education. Among the individual indicators of frailty, missingness ranged from 0.1% to 1.9%.

^b^ Categories represent a combination of race and ethnicity as self-reported in the NHATS interview. The category Hispanic includes participants of all races who reported Hispanic ethnicity; other includes participants reporting race as American Indian, Alaska Native, Asian, Native Hawaiian, Pacific Islander, or other race.

^c^ Range, 0 to 9.

^d^ The frailty score is derived from the composite of 1 point for each of the 5 frailty criteria (range, 0-5): weight loss, muscle weakness, exhaustion, slow gait speed, and low physical activity.

^e^ Count of disabilities was characterized as the need for help or inability to perform 4 activities of daily living (eating, bathing, using the toilet, and dressing) and 3 mobility activities (getting outside, getting around inside one’s home, getting out of bed) (range, 0-7).

^f^ Ascertained from hospitalization record in linked Medicare claims data.

^g^ Ascertained from linked Medicare claims data using *International Classification of Diseases, Ninth Revision, Clinical Modification* (*ICD-9-CM*) (96.7X) and *International Statistical Classification of Diseases, Tenth Revision, Procedure Coding System* (*ICD-10-PCS*) (5A1935Z,5A1945Z,5A1955Z) codes for mechanical ventilation.

A total of 1076 patients (68.5%) received any PT or OT during ICU hospitalizations. We observed decreased receipt of any PT or OT for hospitalizations among patients with Medicare and Medicaid dual eligibility (228 hospitalizations [62.7%] vs 848 hospitalizations among patients without dual eligibility [69.9%]), rural residence (185 hospitalizations [60.1%] vs 891 hospitalizations among patients from urban areas [70.3%]), and below-median income (520 hospitalizations [65.5%] vs 586 hospitalizations among patients with above-median income [70.8%]). A mean of 0.94 (95% CI, 0.86-1.02) units/d was delivered. Patients with LEP received a lower rate of PT or OT (0.79 [95% CI, 0.76- 0.82] units/d vs 0.95 [95% CI, 0.90-0.99] units/d for those without LEP), as did patients with income above the median (0.84 [95% CI, 0.73-0.96] units/d vs 1.02 [95% CI, 0.91-1.03] units/d for those below median income).

**Figure 1.**
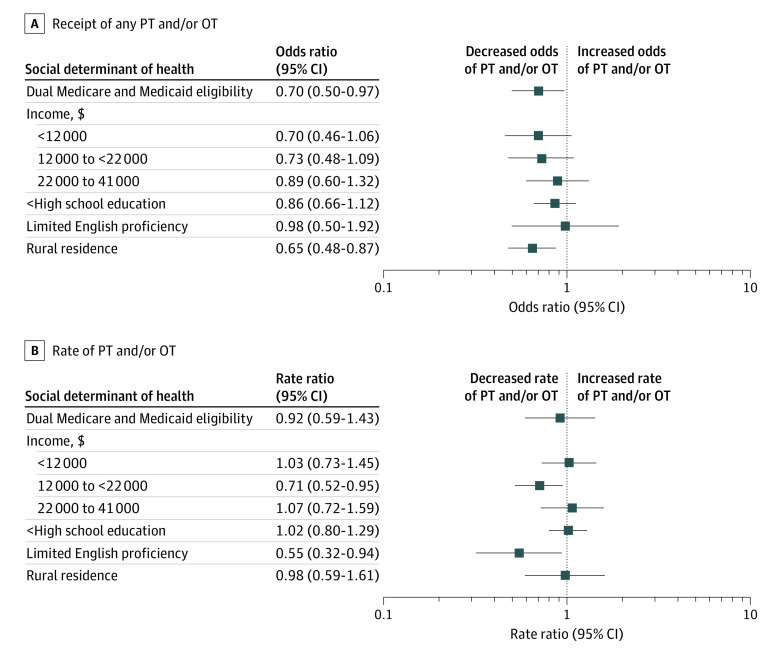
Associations of Social Determinants of Health With Delivery of Physical Therapy (PT) or Occupational Therapy (OT) During Intensive Care Unit Hospitalization A, Adjusted odds ratios and 95% CIs for the outcome of any in-hospital PT or OT were derived from logistic regression models. B, Adjusted rate ratios and 95% CIs for the outcome of rate of in-hospital PT or OT were derived from Poisson regression models. All models were constructed separately for each exposure and adjusted for the same set of covariates (age, sex, pre–intensive care unit count of disabilities in activities of daily living, use of mechanical ventilation, and count of organ dysfunction as a surrogate for severity of illness).

**Figure 2.**
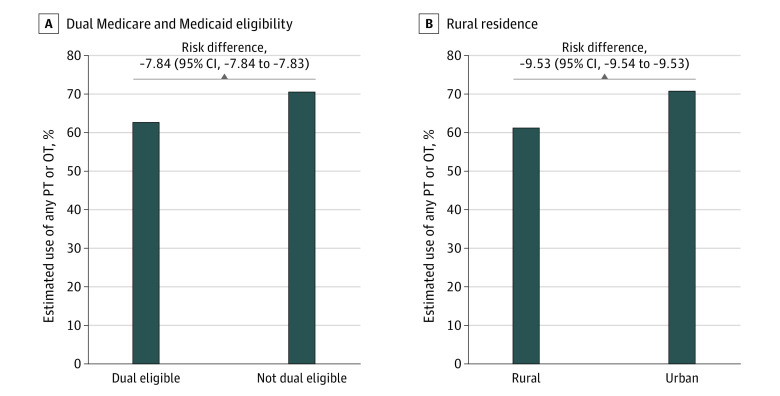
Absolute Risk Differences in the Adjusted Percentages of Intensive Care Unit Hospitalizations With Delivery of Any Physical Therapy (PT) or Occupational Therapy (OT) Estimates were derived from the multivariable logistic regression model adjusting for covariates of age, sex, pre–intensive care unit count of disabilities in activities of daily living, use of mechanical ventilation, and count of organ dysfunction as a surrogate for severity of illness.

**Figure 3.**
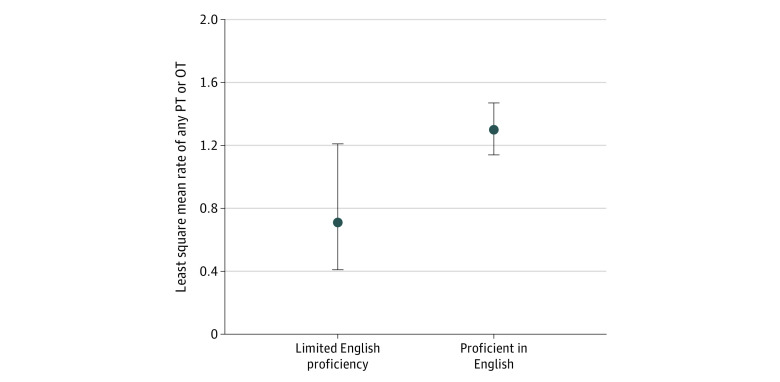
Least Square Means of Rate of Physical Therapy (PT) or Occupational Therapy (OT) Delivered During Intensive Care Unit Hospitalization Among Patients With Limited English Proficiency Estimates were derived from the multivariable Poisson regression model adjusting for covariates of age, sex, pre–intensive care unit count of disabilities in activities of daily living, use of mechanical ventilation, and count of organ dysfunction as a surrogate for severity of illness, as described in the methods.

Figure 1 presents the results of our multivariable models. Dual Medicare and Medicaid eligibility (adjusted odds ratio [aOR], 0.70 [95% CI, 0.50-0.97]) and rural residence (aOR, 0.65 [95% CI, 0.48-0.87]) were associated with lower odds of delivery of any PT or OT (Figure 1A). For risk differences, the percentage of ICU hospitalizations in which any PT or OT was delivered was 7.8% lower for dual-eligible older adults than for non-dual-eligible older adults and 9.5% lower for residents of rural vs urban areas (Figure 2). For the rate of total therapy, LEP was associated with lower rates of in-hospital PT or OT compared with not having LEP (adjusted rate ratio [aRR], 0.55 [95% CI, 0.32-0.94]) (Figure 1B). On the absolute scale, a mean rate of 0.7 (95% CI, 0.4-1.2) units/d of PT or OT was delivered to participants with LEP, compared with 1.3 (95% CI, 1.1-1.5) units/d to those proficient in English (Figure 3). Over a 5-day hospital stay, this would translate into 24 to 66 fewer minutes of therapy for patients with LEP compared with those proficient in English. Having an income between $12 000 and $22 000 was also significantly associated with a lower rate of therapy delivered (aRR, 0.71 [95% CI, 0.52-0.95]) compared with the highest quartile of income; the other income categories did not demonstrate significant associations (Figure 1B).

In sensitivity analyses excluding hospitalization for participants who were admitted from a nursing home or had a nursing home stay of at least 100 days between their pre-ICU NHATS interview and the index ICU hospitalization, the magnitude and direction of association between the exposures and both outcomes were similar, albeit with wider CIs (eTable 2 and eTable 3 in Supplement 1).

## Discussion

In this nationally representative cohort study of older adults, we found that select SDOH were associated with reduced delivery of skilled rehabilitation services during hospitalization for critical illness. After accounting for prehospitalization disability and severity of acute illness, older adults who were dually eligible for Medicare and Medicaid and those who resided in rural areas had 30% to 35% lower odds of receiving any PT or OT during an ICU hospitalization than patients who were not dually eligible or who were proficient in English. Patients with LEP received lower amounts of therapy than patients proficient in English. Given the well-documented value of in-hospital rehabilitative PT and OT in preventing functional decline and identifying care needs at discharge following critical illness,^6,7,8,9^ our findings of reduced delivery of these services to older adults by dual eligibility status, rural residence, and LEP warrant consideration of targeted efforts to mitigate inequities.

The prevalence of in-hospital rehabilitation delivery in our study was comparable with estimates from contemporary studies of acutely hospitalized patients.^28,37,38^ Among patients who are critically ill, clinician surveys^11,12,39^ and cohort studies of hospitals participating in trials or quality reporting initiatives^14,16^ have reported wide variability in the use of PT and OT. Despite this known variability, to our knowledge, prior work has not evaluated the role of SDOH in the delivery of skilled rehabilitation services among Medicare beneficiaries. The availability of patient-level information on SDOH in NHATS, beyond those usually available in administrative data, allowed us to evaluate this important question.

Our findings of reduced delivery of skilled rehabilitation to older adults who are dually eligible for Medicare and Medicaid, live in rural areas, or have LEP may have explanations rooted in structural- and individual-level factors underlying in-hospital care delivery. First, factors related to resources and organization of rehabilitation services at hospitals deserve consideration. Staffing by physical therapists and nurses is associated with rehabilitation among patients who are critically ill.^11,18^ Hospitals serving more dually eligible patients and those located in rural areas are generally more underresourced and may not be adequately staffed by professionals essential to delivering rehabilitation.^40^ Similar to our findings, dual Medicare and Medicaid eligibility was associated with a lower likelihood of skilled rehabilitation among patients with acute stroke.^28^ Second, percolation of evidence-based strategies, such as the ABCDEF bundle (assess, prevent, and manage pain; both spontaneous awakening and spontaneous breathing trials; choice of analgesia and sedation; assess, prevent, and manage delirium; early mobility and exercise; and family engagement and empowerment),^6,41^ to promote rehabilitation may be lower at these hospitals. Study collaboratives promoting adoption of the bundle have typically engaged urban, academic hospitals^15^; whether and how this guidance is translated into practice in rural hospitals should be explored. Third, the association between LEP and reduced PT and OT suggests that interpersonal communication barriers or implicit biases can underlie suboptimal delivery of rehabilitation, as described for other care processes.^20,42^ Consistent with our observations, in a 2020 study at a safety-net hospital in Texas, speaking a language other than English or Spanish was associated with fewer minutes of therapy delivered to older adults hospitalized with prolonged acute illness.^29^ Among patients who are critically ill, the reduced amount of rehabilitation services delivered to those with LEP could be due to differential prescription of rehabilitation services by physicians because of perceived barriers to engagement or differences in management of sedation and delirium that might influence a patient’s ability to participate in rehabilitation.^17,19,20^

Our findings have important implications. First, immobility, a common occurrence during ICU hospitalization, is an important and modifiable risk factor for post-ICU disability.^6,7,8^ While mobilizing hospitalized patients who are acutely ill can be achieved by personnel other than rehabilitation therapists, the complex feasibility and safety considerations for older adults who are critically ill usually warrant an interdisciplinary approach.^13,43,44^ Evaluation by physical or occupational therapists is a part of recommended ICU mobilization protocols^6,7,45^ and associated with its delivery in observational studies.^13,14^ Therefore, while not equivalent to it, the lower delivery of PT or OT services by SDOH in our study likely represents underlying differences in ICU mobilization. Second, evaluation by physical and occupational therapists, usually on the wards, is important to identify postacute rehabilitation needs before hospital discharge. Reduced delivery of any PT or OT during hospitalization suggests that postacute rehabilitation needs are assessed less frequently among older adults with Medicaid and those in rural areas. In-hospital skilled rehabilitation is associated not only with higher mobility at discharge^9,46,47^ but also improved long-term function among adults recovering from critical illness.^48^ Therefore, reduced delivery of any rehabilitation during ICU hospitalization may represent a missed opportunity to improve long-term functional outcomes after critical illness and inequities in this practice can be a mechanism underlying disparities in post-ICU disability.^10^

Our study has several strengths. First, we used a nationally representative sample of older adults with ICU hospitalization. Second, we included granular assessment of prehospitalization disability that could influence in-hospital rehabilitation needs. Third, we had information on SDOH using instruments specifically tailored for older adults,^23^ Fourth, we used rigorous methods to determine delivery of PT and OT using claims data.^28,49,50^

### Limitations

Our findings should be interpreted in the context of a few limitations. First, we could not distinguish the contribution of hospital-level effects or evaluate hospital factors, such as supply of physical therapists, due to the limited number of observations per hospital and hospital-level information in our claims-linked NHATS data. This should be investigated in future work to understand the role of structural factors in driving differences in rehabilitation delivery. Second, we could not distinguish the delivery of PT and OT in the ICU from that delivered elsewhere in the hospital; future studies evaluating this could guide efforts to mitigate differences. Third, we could not evaluate nurse-driven mobility. While this practice exists, an interdisciplinary approach, including at least an evaluation by rehabilitation therapists, remains usual practice in most US ICUs,^13,44^ suggesting that in-hospital skilled rehabilitation, although not a surrogate for ICU mobilization, is likely associated with differences in this practice. Fourth, we did not have information on severity of illness scores, such as the SOFA score and Acute Physiology and Chronic Health Evaluation scale score, which are known to be associated with rehabilitation in the ICU.^51^ Nevertheless we used a validated claims-based organ dysfunction algorithm to account for severity of illness.^27^ Fifth, because of the small number of participants with self-reported race and ethnicity other than non-Hispanic Black or non-Hispanic White in our sample, we could not evaluate it as an exposure; future work should investigate this. Furthermore, because LEP and rurality were evaluated as exploratory exposures in our study, the observed associations should be further investigated in future studies.

## Conclusions

In this nationally representative cohort study of older adults, dual eligibility for Medicare and Medicaid and rural residency were associated with lower likelihood and LEP was associated with reduced rate of delivery of skilled rehabilitation therapy during hospitalization with critical illness. Our findings highlight the need to consider these SDOH in efforts to enhance equitable delivery of skilled rehabilitation services during hospitalization. Future research is needed to distinguish individual- vs structural-level factors underlying differences in in-hospital rehabilitation delivery by SDOH.

## Notes

Supplement 1. **eFigure.** Assembly of the Analytic Sample **eTable 1.** ICD-9 and ICD-10 Codes Used to Identify Mechanical Ventilation and Organ Dysfunction **eTable 2.** Sensitivity Analysis for the Outcome of Any In-Hospital Physical and/or Occupational Therapy Excluding ICU Hospitalizations From NHATS Participants Who Were Admitted From a Nursing Home or Had a Stay in a Nursing Home of ≥100 Days Between Pre-ICU NHATS Interview and Index ICU Hospitalization **eTable 3.** Sensitivity Analysis for the Outcome of Rate of In-Hospital Physical and/or Occupational Therapy Excluding ICU Hospitalizations From NHATS Participants Who Were Admitted From a Nursing Home or Had a Stay in a Nursing Home of ≥100 Days Between Pre-ICU NHATS Interview and Index ICU Hospitalization

Supplement 2.
Data Sharing Statement

## References

[1] Wunsch H, Guerra C, Barnato AE, Angus DC, Li G, Linde-Zwirble WT. Three-year outcomes for Medicare beneficiaries who survive intensive care. JAMA. 2010;303(9):849-856. doi:10.1001/jama.2010.216 20197531

[2] Iwashyna TJ, Cooke CR, Wunsch H, Kahn JM. Population burden of long-term survivorship after severe sepsis in older Americans. J Am Geriatr Soc. 2012;60(6):1070-1077. doi:10.1111/j.1532-5415.2012.03989.x 22642542 PMC3374893

[3] Needham DM, Bronskill SE, Calinawan JR, Sibbald WJ, Pronovost PJ, Laupacis A. Projected incidence of mechanical ventilation in Ontario to 2026: preparing for the aging baby boomers. Crit Care Med. 2005;33(3):574-579. doi:10.1097/01.CCM.0000155992.21174.31 15753749

[4] Iwashyna TJ, Ely EW, Smith DM, Langa KM. Long-term cognitive impairment and functional disability among survivors of severe sepsis. JAMA. 2010;304(16):1787-1794. doi:10.1001/jama.2010.1553 20978258 PMC3345288

[5] Ferrante LE, Pisani MA, Murphy TE, Gahbauer EA, Leo-Summers LS, Gill TM. Functional trajectories among older persons before and after critical illness. JAMA Intern Med. 2015;175(4):523-529. doi:10.1001/jamainternmed.2014.7889 25665067 PMC4467795

[6] Devlin JW, Skrobik Y, Gélinas C, . Executive summary: clinical practice guidelines for the prevention and management of pain, agitation/sedation, delirium, immobility, and sleep disruption in adult patients in the ICU. Crit Care Med. 2018;46(9):1532-1548. doi:10.1097/CCM.0000000000003259 30113371

[7] Schweickert WD, Pohlman MC, Pohlman AS, . Early physical and occupational therapy in mechanically ventilated, critically ill patients: a randomised controlled trial. Lancet. 2009;373(9678):1874-1882. doi:10.1016/S0140-6736(09)60658-9 19446324 PMC9906655

[8] Anekwe DE, Biswas S, Bussières A, Spahija J. Early rehabilitation reduces the likelihood of developing intensive care unit-acquired weakness: a systematic review and meta-analysis. Physiotherapy. 2020;107:1-10. doi:10.1016/j.physio.2019.12.004 32135387

[9] Naylor MD, Brooten D, Campbell R, . Comprehensive discharge planning and home follow-up of hospitalized elders: a randomized clinical trial. JAMA. 1999;281(7):613-620. doi:10.1001/jama.281.7.613 10029122

[10] Jain S, Murphy TE, O’Leary JR, Leo-Summers L, Ferrante LE. Association between socioeconomic disadvantage and decline in function, cognition, and mental health after critical illness among older adults: a cohort study. Ann Intern Med. 2022;175(5):644-655. doi:10.7326/M21-3086 35254879 PMC9316386

[11] Jolley SE, Dale CR, Hough CL. Hospital-level factors associated with report of physical activity in patients on mechanical ventilation across Washington State. Ann Am Thorac Soc. 2015;12(2):209-215. doi:10.1513/AnnalsATS.201410-480OC 25565021 PMC4342832

[12] Hodgin KE, Nordon-Craft A, McFann KK, Mealer ML, Moss M. Physical therapy utilization in intensive care units: results from a national survey. Crit Care Med. 2009;37(2):561-566. doi:10.1097/CCM.0b013e3181957449 19114903 PMC2908523

[13] Bakhru RN, Wiebe DJ, McWilliams DJ, Spuhler VJ, Schweickert WD. An environmental scan for early mobilization practices in U.S. ICUs. Crit Care Med. 2015;43(11):2360-2369. doi:10.1097/CCM.0000000000001262 26308435

[14] Jolley SE, Moss M, Needham DM, ; Acute Respiratory Distress Syndrome Network Investigators. Point prevalence study of mobilization practices for acute respiratory failure patients in the United States. Crit Care Med. 2017;45(2):205-215. doi:10.1097/CCM.0000000000002058 27661864 PMC5520580

[15] Balas MC, Tan A, Pun BT, . Effects of a national quality improvement collaborative on ABCDEF bundle implementation. Am J Crit Care. 2022;31(1):54-64. doi:10.4037/ajcc2022768 34972842 PMC9972543

[16] Prohaska CC, Sottile PD, Nordon-Craft A, . Patterns of utilization and effects of hospital-specific factors on physical, occupational, and speech therapy for critically ill patients with acute respiratory failure in the USA: results of a 5-year sample. Crit Care. 2019;23(1):175. doi:10.1186/s13054-019-2467-9 31097017 PMC6524324

[17] Potter K, Miller S, Newman S. Patient-level barriers and facilitators to early mobilization and the relationship with physical disability post-intensive care: part 2 of an integrative review through the lens of the World Health Organization *International Classification of Functioning, Disability, and Health*. Dimens Crit Care Nurs. 2021;40(3):164-173. doi:10.1097/DCC.0000000000000470 33792276

[18] Potter K, Miller S, Newman S. Environmental factors affecting early mobilization and physical disability post-intensive care: an integrative review through the lens of the World Health Organization *International Classification of Functioning, Disability, and Health*. Dimens Crit Care Nurs. 2021;40(2):92-117. doi:10.1097/DCC.0000000000000461 33961378

[19] Parry SM, Knight LD, Connolly B, . Factors influencing physical activity and rehabilitation in survivors of critical illness: a systematic review of quantitative and qualitative studies. Intensive Care Med. 2017;43(4):531-542. doi:10.1007/s00134-017-4685-4 28210771

[20] Gershengorn HB, Patel S, Mallow CM, . Association of language concordance and restraint use in adults receiving mechanical ventilation. Intensive Care Med. 2023;49(12):1489-1498. doi:10.1007/s00134-023-07243-0 37843570

[21] Lloren A, Liu S, Herrin J, . Measuring hospital-specific disparities by dual eligibility and race to reduce health inequities. Health Serv Res. 2019;54(Suppl 1)(suppl 1):243-254. doi:10.1111/1475-6773.13108 30666634 PMC6341208

[22] Bahiru E, Ziaeian B, Moucheraud C, . Association of dual eligibility for Medicare and Medicaid with heart failure quality and outcomes among Get With The Guidelines—Heart Failure hospitals. JAMA Cardiol. 2021;6(7):791-800. doi:10.1001/jamacardio.2021.0611 33825802 PMC8027938

[23] Kasper JD, Freedman VA. National Health and Aging Trends Study (NHATS): user guide. Accessed July 28, 2023. https://nhats.org/sites/default/files/2021-01/NHATS_Round_1_User_Guide_Final_Release_0.pdf

[24] Montaquila J, Freedman VA, Edwards B, Kasper JD. National Health and Aging Trends Study (NHATS): round 1 sample design and selection. Accessed July 28, 2023. https://www.nhats.org/sites/default/files/2021-01/NHATS%20Round%201%20Sample%20Design%2005_10_12_2.pdf

[25] Sjoding MW, Prescott HC, Wunsch H, Iwashyna TJ, Cooke CR. Longitudinal changes in ICU admissions among elderly patients in the United States. Crit Care Med. 2016;44(7):1353-1360. doi:10.1097/CCM.0000000000001664 26968023 PMC4911310

[26] Quan H, Parsons GA, Ghali WA. Validity of procedure codes in *International Classification of Diseases, 9th Revision,* clinical modification administrative data. Med Care. 2004;42(8):801-809. doi:10.1097/01.mlr.0000132391.59713.0d 15258482

[27] Bosch NA, Law AC, Rucci JM, Peterson D, Walkey AJ. Predictive validity of the Sequential Organ Failure Assessment Score versus claims-based scores among critically ill patients. Ann Am Thorac Soc. 2022;19(6):1072-1076. doi:10.1513/AnnalsATS.202111-1251RL 35266866 PMC9797032

[28] Kumar A, Adhikari D, Karmarkar A, . Variation in hospital-based rehabilitation services among patients with ischemic stroke in the United States. Phys Ther. 2019;99(5):494-506. doi:10.1093/ptj/pzz014 31089705 PMC6489167

[29] Nguyen DQ, Ifejika NL, Reistetter TA, Makam AN. Factors associated with duration of rehabilitation among older adults with prolonged hospitalization. J Am Geriatr Soc. Published online December 22, 2020. doi:10.1111/jgs.16988 33393088 PMC8217402

[30] Falvey JR, Murphy TE, Gill TM, Stevens-Lapsley JE, Ferrante LE. Home health rehabilitation utilization among Medicare beneficiaries following critical illness. J Am Geriatr Soc. 2020;68(7):1512-1519. doi:10.1111/jgs.16412 32187664 PMC7712590

[31] Albrecht JS, Kumar A, Falvey JR. Association Between race and receipt of home- and community-based rehabilitation after traumatic brain injury among older Medicare beneficiaries. JAMA Surg. 2023;158(4):350-358. doi:10.1001/jamasurg.2022.7081 36696119 PMC9878433

[32] Montaquila J, Freedman VA, Kasper JD. National Health and Aging Trends Study (NHATS): round 1 income imputation. Accessed July 28, 2023. https://www.nhats.org/sites/default/files/2021-01/NHATS_Round1_Income_Imputation_11_09_12.pdf

[33] Franco Y, Choi EY. The relationship between immigrant status and undiagnosed dementia: the role of limited English proficiency. J Immigr Minor Health. 2020;22(5):914-922. doi:10.1007/s10903-019-00963-w 31893329

[34] Falvey JR, Cohen AB, O’Leary JR, Leo-Summers L, Murphy TE, Ferrante LE. Association of social isolation with disability burden and 1-year mortality among older adults with critical illness. JAMA Intern Med. 2021;181(11):1433-1439. doi:10.1001/jamainternmed.2021.5022 34491282 PMC8424527

[35] Freedman VA, Hu M, DeMatteis J, Kasper JD. Accounting for sample design in NHATS and NSOC analyses: frequently asked questions. Accessed July 28, 2023. https://www.nhats.org/sites/default/files/2021-01/Accounting_for_the_NHATS_NSOC_Design_in_Analyses_FAQ_0.pdf

[36] Montaquila J, Freedman VA, Spillman B, Kasper JD. National Health and Aging Trends Study (NHATS): development of round 1 survey weights. Accessed July 28, 2023. https://www.nhats.org/sites/default/files/2021-01/NHATS%20Round%201%20Weighting%20Description_Nov2012_3.pdf

[37] Freburger JK, Chou A, Euloth T, Matcho B. Variation in Acute Care Rehabilitation and 30-Day Hospital Readmission or Mortality in Adult Patients With Pneumonia. JAMA Netw Open. 2020;3(9):e2012979. doi:10.1001/jamanetworkopen.2020.12979 32886119 PMC7489809

[38] Rauzi MR, Ridgeway KJ, Wilson MP, . Rehabilitation Therapy Allocation and Changes in Physical Function Among Patients Hospitalized Due to COVID-19: A Retrospective Cohort Analysis. Phys Ther. 2023;103(3):pzad007. doi:10.1093/ptj/pzad007 37172130 PMC10071586

[39] Bakhru RN, McWilliams DJ, Wiebe DJ, Spuhler VJ, Schweickert WD. Intensive Care Unit Structure Variation and Implications for Early Mobilization Practices. An International Survey. Ann Am Thorac Soc. 2016;13(9):1527-1537. doi:10.1513/AnnalsATS.201601-078OC 27268952 PMC5059498

[40] Institute of Medicine. America’s Health Care Safety Net: Intact But Endangered. National Academies Press; 2000. 25077222

[41] Pun BT, Balas MC, Barnes-Daly MA, . Caring for critically ill patients with the ABCDEF bundle: results of the ICU Liberation Collaborative in over 15,000 adults. Crit Care Med. 2019;47(1):3-14. doi:10.1097/CCM.0000000000003482 30339549 PMC6298815

[42] Pérez-Stable EJ, El-Toukhy S. Communicating with diverse patients: How patient and clinician factors affect disparities. Patient Educ Couns. 2018;101(12):2186-2194. doi:10.1016/j.pec.2018.08.021 30146407 PMC6417094

[43] Schallom M, Tymkew H, Vyers K, . Implementation of an interdisciplinary AACN early mobility protocol. Crit Care Nurse. 2020;40(4):e7-e17. doi:10.4037/ccn2020632 32737495

[44] Rawal H, Bakhru RN. Early mobilization in the ICU. CHEST Crit Care. 2023;2(1):100038. doi:10.1016/j.chstcc.2023.100038

[45] Morris PE, Goad A, Thompson C, . Early intensive care unit mobility therapy in the treatment of acute respiratory failure. Crit Care Med. 2008;36(8):2238-2243. doi:10.1097/CCM.0b013e318180b90e 18596631

[46] Johnson JK, Lapin B, Green K, Stilphen M. Frequency of Physical therapist intervention is associated with mobility status and disposition at hospital discharge for patients with COVID-19. Phys Ther. 2021;101(1):pzaa181. doi:10.1093/ptj/pzaa181 32986836 PMC7543647

[47] Tipping CJ, Harrold M, Holland A, Romero L, Nisbet T, Hodgson CL. The effects of active mobilisation and rehabilitation in ICU on mortality and function: a systematic review. Intensive Care Med. 2017;43(2):171-183. doi:10.1007/s00134-016-4612-0 27864615

[48] Morris PE, Berry MJ, Files DC, . Standardized rehabilitation and hospital length of stay among patients with acute respiratory failure: a randomized clinical trial. JAMA. 2016;315(24):2694-2702. doi:10.1001/jama.2016.7201 27367766 PMC6657499

[49] Capo-Lugo CE, Askew RL, Boebel M, DeLeo C, Deutsch A, Heinemann A. A comparative approach to quantifying provision of acute therapy services. Medicine (Baltimore). 2021;100(40):e27377. doi:10.1097/MD.0000000000027377 34622841 PMC8500582

[50] Jette DU, Brown R, Collette N, Friant W, Graves L. Physical therapists’ management of patients in the acute care setting: an observational study. Phys Ther. 2009;89(11):1158-1181. doi:10.2522/ptj.20080338 19729390

[51] Mendez-Tellez PA, Dinglas VD, Colantuoni E, . Factors associated with timing of initiation of physical therapy in patients with acute lung injury. J Crit Care. 2013;28(6):980-984. doi:10.1016/j.jcrc.2013.06.001 23845792 PMC3830674

