# Supplementary material for: Social Determinants of Health and Delivery of Rehabilitation to Older Adults During ICU Hospitalization
Source: JAMA Netw Open. 2024 May 10;7(5):e2410713. doi: 10.1001/jamanetworkopen.2024.10713 (PMC11087837; doi:10.1001/jamanetworkopen.2024.10713)
Supplement: Supplement 1. — eFigure. Assembly of the Analytic Sample eTable 1. ICD-9 and ICD-10 Codes Used to Identify Mechanical Ventilation and Organ Dysfunction eTable 2. Sensitivity Analysis for the Outcome of Any In-Hospital Physical and/or Occupational Therapy Excluding ICU Hospitalizations From NHATS Participants Who Were Admitted From a Nursing Home or Had a Stay in a Nursing Home of ≥100 Days Between Pre-ICU NHATS Interview and Index ICU Hospitalization eTable 3. Sensitivity Analysis for the Outcome of Rate of In-Hospital Physical and/or Occupational Therapy Excluding ICU Hospitalizations From NHATS Participants Who Were Admitted From a Nursing Home or Had a Stay in a Nursing Home of ≥100 Days Between Pre-ICU NHATS Interview and Index ICU Hospitalization [file jamanetwopen-e2410713-s001.pdf]

Supplemental Online Content

Jain S, Murphy TE, Falvey JR, et al. Social determinants of health and delivery of rehabilitation to older adults during ICU hospitalization. *JAMA Netw Open*. 2024;7(5):e2410713. doi:10.1001/jamanetworkopen.2024.10713

eFigure. Assembly of the Analytic Sample
eTable 1. *ICD-9* and *ICD-10* Codes Used to Identify Mechanical Ventilation and Organ Dysfunction
eTable 2. Sensitivity Analysis for the Outcome of Any In-Hospital Physical and/or Occupational Therapy Excluding ICU Hospitalizations From NHATS Participants Who Were Admitted From a Nursing Home or Had a Stay in a Nursing Home of ≥100 Days Between Pre-ICU NHATS Interview and Index ICU Hospitalization
eTable 3. Sensitivity Analysis for the Outcome of Rate of In-Hospital Physical and/or Occupational Therapy Excluding ICU Hospitalizations From NHATS Participants Who Were Admitted From a Nursing Home or Had a Stay in a Nursing Home of ≥100 Days Between Pre-ICU NHATS Interview and Index ICU Hospitalization

This supplemental material has been provided by the authors to give readers additional information about their work.

© 2024 Jain S et al. *JAMA Network Open.*

eFigure. Assembly of the Analytic Sample.

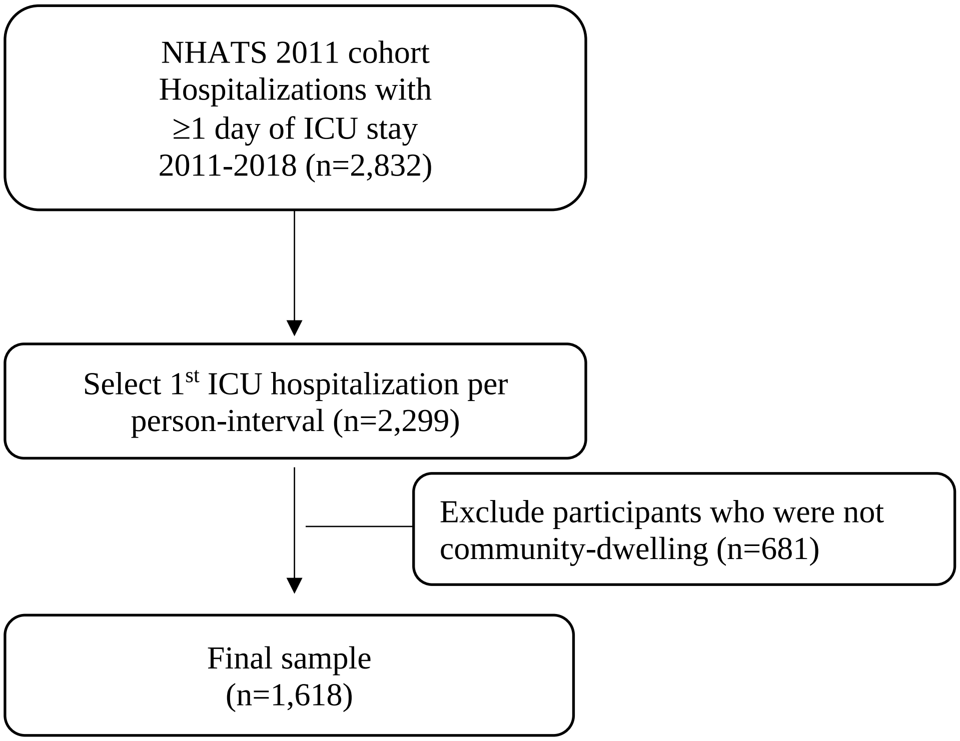

© 2024 Jain S et al. *JAMA Network Open.*

eTable 1. ICD-9-CM and ICD-10-CM codes used to identify mechanical ventilation and organ dysfunction.

Organ System	ICD-9 CM	ICD-10 CM
Circulatory	785.5, 458	R57.x (R57.0, R57.1, R57.8, R57.9), R65.21, I95.1-3, I95.89-9, I95.81
Renal	584.x	N17.x
Neurologic	348.3, 293, 348.1	F05, G93.1, G93.4x, F06.x, F53, I67.83
Hematologic	287.4, 287.5, 286.9, 286.6	D65, D68.8, D68.9, D69.51, D69.59-6
Hepatic	570, 573.4	K72.00-01, K76.2, K76.3
Mechanical Ventilation	96.7x	5A1935Z, 5A1945Z, 5A1955Z

© 2024 Jain S et al. *JAMA Network Open.*

eTable2. Sensitivity analysis for the outcome of any in-hospital physical and/or occupational therapy excluding ICU hospitalizations from NHATS participants who were admitted from a nursing home or had a stay in a nursing home of ≥ 100 days between pre-ICU NHATS interview and index ICU hospitalization (n = 1,506).

**Social Determinant of Health**	**Adjusted Odds Ratio (95% C.I.)**
Dual eligibility for Medicare and Medicaid	0.73 (0.51, 1.04)
Rural residence (vs urban)	0.65 (0.48, 0.88)
Race (non-White vs White)	1.00 (0.74, 1.35)
Income	
Quartile 1 (< $12,000)	0.76 (0.50, 1.16)
Quartile 2 ($12,000 - $22,000)	0.76 (0.49, 1.17)
Quartile 3 ($22,001 - $41,000)	0.93 (0.62, 1.39)
Less than high school education (vs more)	0.87 (0.66, 1.15)
Limited English Proficiency (vs no Limited English Proficiency)	0.95 (0.49, 1.85)

*All estimates were derived from multivariable logistic regression models with the outcome of delivery of any physical and/or occupational therapy and covariates of age, sex, pre-ICU count of disabilities in activities of daily living, use of mechanical ventilation, and count of organ dysfunction, as described in the methods.

© 2024 Jain S et al. *JAMA Network Open.*

eTable3. Sensitivity analysis for the outcome of rate of in-hospital physical and/or occupational therapy excluding ICU hospitalizations from NHATS participants who were admitted from a nursing home or had a stay in a nursing home of ≥ 100 days between pre-ICU NHATS interview and index ICU hospitalization (n = 1,506).

**Social Determinant**	**Adjusted Rate Ratio (95% C.I.)**
Dual eligibility for Medicare and Medicaid	0.95 (0.60, 1.51)
Rural residence (vs urban)	0.98 (0.59, 1.61)
Race (non-White vs White)	0.98 (0.70, 1.38)
Income (Referent to income > $41,000)	
Quartile 1 (< $12,000)	1.09 (0.77, 1.55)
Quartile 2 ($12,000 - $22,000)	0.73 (0.53, 0.99)
Quartile 3 ($22,001 - $41,000)	1.12 (0.74, 1.69)
Less than high school education (vs more)	1.04 (0.81, 1.33)
Limited English Proficiency (vs no Limited English Proficiency)	0.53 (0.30, 0.95)

All estimates were derived from multivariable Poisson regression models with the outcome of delivery of any physical and/or occupational therapy and covariates of age, sex, pre-ICU count of disabilities in activities of daily living, use of mechanical ventilation, and count of organ dysfunction, as described in the methods.

© 2024 Jain S et al. *JAMA Network Open.*
