# Supplementary material for: Social Determinants of Health and Delivery of Rehabilitation to Older Adults During ICU Hospitalization
Source: JAMA Netw Open. 2024 May 10;7(5):e2410713. doi: 10.1001/jamanetworkopen.2024.10713 (PMC11087837; doi:10.1001/jamanetworkopen.2024.10713)
Supplement: Supplement 2. — Data Sharing Statement [file jamanetwopen-e2410713-s002.pdf]

Data Sharing Statement

Jain. Social Determinants of Health and Delivery of Rehabilitation to Older Adults During ICU Hospitalization. *JAMA Netw Open*. Published May 10, 2024.
doi:10.1001/jamanetworkopen.2024.10713

Data
Data available: Yes
Data types: Data dictionary
How to access data: The NHATS-linked Medicare data for restricted use is made available by MedRIC and Johns Hopkins University and requires a Data Use Agreement with both these institutions. We will make available the dictionary of data fields of these secondary adat used in our study, however, interested readers will need to contact the aforementioned agencies for access to the unidentified data.
When available: With publication

Supporting Documents
Document types: None

Additional Information
Who can access the data: To researchers whose proposed use of the data has been approved
Types of analyses: For a specified purpose
Mechanisms of data availability: After approval of a proposal
